# Complete Genome Sequences of Two Flavobacterium ammonificans Strains and a Flavobacterium ammoniigenes Strain of Ammonifying Bacterioplankton Isolated from Surface River Water

**DOI:** 10.1128/mra.00176-22

**Published:** 2022-06-06

**Authors:** Wataru Suda, Yusuke Ogata, Chie Shindo, Keiji Watanabe

**Affiliations:** a RIKEN Center for Integrative Medical Sciences, Yokohama, Kanagawa, Japan; b Center for Environmental Science in Saitama, Kazo, Saitama, Japan; University of Delaware

## Abstract

Flavobacterium ammonificans and Flavobacterium ammoniigenes are ammonifying freshwater bacterioplankton. Here, we report the complete genome sequences of two F. ammonificans strains (SHINM13^T^ and GENT11) and one F. ammoniigenes strain (GENT5^T^) that were isolated from surface river water in Japan.

## ANNOUNCEMENT

Flavobacterium ammonificans and Flavobacterium ammoniigenes belong to the family *Flavobacteriaceae* (order *Flavobacteriales*, class *Flavobacteriia*, phylum *Bacteroidetes*). This taxonomic assignment was determined using a combination of phenotypic (e.g., respiratory quinones, fatty acids, and polar lipids) and genotypic (e.g., 16S rRNA gene and genome phylogenies, average nucleotide identity [ANI], digital DNA-DNA hybridization [dDDH], and average amino acid identity [AAI]) characteristics (1). These isolates convert dissolved organic nitrogen to ammonium (a process called ammonification) during cell growth (1).

Here, we report the complete genome sequences of two F. ammonificans strains, SHINM13^T^ (JCM 34684^T^ = NCIMB 15379^T^) and GENT11 (JCM 34685), and an F. ammoniigenes strain, GENT5^T^ (JCM 32249^T^ = NCIMB 15380^T^), that were isolated from surface river water samples collected from the Koyama River, Fukaya, Saitama, Japan (36°13′50.8″N, 139°18′31.6″E, and 36°10′03.0″N, 139°06′34.9″E) on 25 April 2013 and 15 May 2014, respectively (1). The samples were filtered through a disposable syringe equipped with a 0.7-μm-particle retention glass fiber filter (Puradisc 25 GF/F disposable filter device; Whatman, Springfield Mill, UK). Filtrates were spread on modified Reasoner’s 2A (MR2A) agar plates and incubated at 25°C for 3 days (2). One bacterial colony for each strain was picked, inoculated into sterilized MR2A liquid medium (pH 7.2), and incubated at 25°C for 2 days with reciprocal shaking (120 rpm). The pure strain cell suspensions were maintained as stocks in MR2A broth supplemented with 20% (wt/vol) glycerol at −80°C for preservation. One glycerol stock for each strain was inoculated and cultivated in MR2A liquid medium, and the cells were harvested by centrifugation for genomic DNA extraction.

The genomic DNA of strains SHINM13^T^, GENT11, and GENT5^T^ was extracted using phenol-chloroform-isoamyl alcohol (3). Whole-genome sequencing of these strains was performed using MiSeq (Illumina, Inc., San Diego, CA, USA) and Sequel II (Pacific Biosciences of California, Inc. [PacBio], Menlo Park, CA, USA) systems. For Illumina and PacBio sequencing, the libraries were prepared using the TruSeq DNA PCR-free library preparation kit (Illumina) with a 550-bp insert size and the SMRTbell template preparation kit v2.0 (PacBio) with DNA shearing by g-TUBE (Covaris, Woburn, MA, USA) with a 10- to 15-kb target length, respectively. The Illumina reads were trimmed and filtered using FASTX-toolkit (v. 0.0.13) (http://hannonlab.cshl.edu/fastx_toolkit), and then the human genome and internal control were removed by mapping with minimap2 (v. 2.13-r850) (3). The PacBio reads were converted to circular consensus sequencing (CCS) reads using the ccs software (v.5.0.0) (https://github.com/PacificBiosciences/ccs). The trimmed Illumina and PacBio CCS reads were assembled using the hybrid assembler Unicycler (v.0.4.8) (4). The obtained genome sequences of the three strains were annotated using DFAST (https://dfast.nig.ac.jp) (5). Default parameters were used for all software unless otherwise specified, and information on the reads obtained and the genome sequences generated is presented in Table 1.

**TABLE 1:** Information on the reads and contigs obtained for Flavobacterium ammonificans strains SHINM13^T^ and GENT11 and F. ammoniigenes strain GENT5^T^

Parameter	Data for strain:		
	SHINM13^T^	GENT11	GENT5^T^
No. of quality-passed MiSeq paired-end reads	888,546	2,558,965	2,258,102
Total size of quality-passed MiSeq paired-end reads (bp)	532,342,750	1,531,421,979	1,351,921,363
Avg length of quality-passed MiSeq paired-end reads (bp)	299.6	299.2	299.3
No. of quality-passed Sequel reads	109,395	3,966	622
Total size of quality-passed Sequel reads (bp)	2,886,229,719	51,443,630	8,169,169
*N*_50_ of quality-passed Sequel reads (bp)	34,030	12,862	13,118
Total no. of contigs	1	1	1
BioProject accession no.	PRJDB12412	PRJDB12412	PRJDB12412
BioSample accession no.	SAMD00409485	SAMD00409483	SAMD00409484
SRA accession no.			
MiSeq reads	DRR321578	DRR321574	DRR321576
Sequel reads	DRR321579	DRR321575	DRR321577
Genome size (bp)	2,409,408	2,271,028	2,263,087
GC content (%)	34.4	34.4	35.1
GenBank/ENA/DDBJ accession no.	AP025185	AP025183	AP025184

Based on the annotation results, we identified that the genomes of strains SHINM13^T^, GENT11, and GENT5^T^ each possessed a gene (*ppk*) predicted to encode polyphosphate kinase, which is associated with the intracellular accumulation of polyphosphate, and a putative xanthorhodopsin-like-protein-encoding gene related to a putative light-driven, proton-pumping rhodopsin (6). Furthermore, the three strains contained putative alanine dehydrogenase-, glutamate dehydrogenase-, and glycine dehydrogenase-like-protein-encoding genes associated with the process of ammonification.

### Data availability.

The chromosome sequences and reads for the two F. ammonificans strains (SHINM13^T^ and GENT11) and the F. ammoniigenes strain (GENT5^T^) were deposited in the GenBank/ENA/DDBJ database, the details of which are presented in Table 1.
